# Bridge to Practice: A Qualitative Evaluation of Joint Medical Program (JMP) International Medical Graduates Perceived Preparedness for Professional Practice

**DOI:** 10.1177/23821205241272360

**Published:** 2024-08-16

**Authors:** Michelle Foot, Khanrin Vashum, Pavana Ballal, Lisa Lampe

**Affiliations:** 1 School of Medicine and Public Health, 5982 University of Newcastle, Callaghan, New South Wales, Australia; 2 Hunter New England (HNE) Health, New Lambton, New South Wales, Australia

**Keywords:** international medical graduates, preparedness, joint medical program, Australia

## Abstract

**OBJECTIVES:**

This study sought to examine the views of international graduates regarding how they perceived the Joint Medical Program Bachelor of Medicine (JMP BMed) undergraduate training prepared them to practice in a health system different from that in which they had studied. Eighteen international graduates of the JMP between 2010 and 2017 inclusive agreed to be interviewed face-to-face.

**METHODS:**

JMP BMed international graduates were interviewed using 18 standardized questions to elicit perceptions of their preparedness to practice and reflections on their training experience. The interview data were qualitatively analyzed, and the main themes were identified and categorized.

**RESULTS:**

Overall, the international graduates of the JMP BMed curriculum felt well-prepared for tasks associated with communication, self-directed learning, and approaching mental health issues. Conversely, they perceived the level of clinical exposure and experience as inadequate in preparing them for the expected level of medical knowledge and responsibility. They also felt underprepared for navigating a different health system.

**CONCLUSIONS:**

The strengths and weaknesses identified in the JMP BMed program in its preparation of international graduates, particularly those who chose to practice in their country of origin, are of relevance for all medical schools that enrol international students. Greater awareness of the needs of international medical students returning home to practice may be of value for future curriculum planning purposes to ensure that medical schools optimally prepare their graduates to practice with confidence in a range of healthcare systems.

## Introduction

Medical education aims to prepare graduates to be knowledgeable, competent, and confident practitioners across a broad range of domains. Newly qualified doctors in their first post-graduate year (PGY1’s) are expected to demonstrate essential competencies and workplace skills as they transition from medical school and integrate into the professional environment in a range of health systems.

In the past few decades, medical education has evolved to integrate innovative teaching, learning, and assessment approaches. One of the most common trends has been the move from traditional, didactic lecture-based learning to problem-based learning (PBL) and case-based learning (CBL) approaches.^1^ The way curricula are conceptualized, the teaching processes that are adopted and the clinical training locations influence the student's experience and the PGY1's perception of preparedness.

Tracking the opinion of graduates about the quality and relevance of their medical school program is important. Globally, medical schools are responsible for training a substantial number of students who are citizens of and/or normally resident in other countries (international students). In Australia, about 1 in 5–6 medical students are international students, according to the Student Statistics Report Australia and New Zealand 2020.^2^ Despite the increasing trend of international students choosing to study in Australia, there is a paucity of literature looking at how well these graduates perform once they join the workforce, particularly in a different health system.^3^

In response to the above, this study was undertaken to gauge how international JMP graduates perceive and compare their level of preparedness when practicing in a system other than the one in which they trained in NSW, Australia. Moreover, this study sought to investigate the impact of the JMP curriculum and obtain feedback identifying shared key experiences; such information can inform future developments in the program. In the JMP, there is a particular focus on communication skills, so we sought to evaluate perceptions of the usefulness of this curriculum element. The JMP also has a strong focus on mental health care and professional self-care. Being aware that this is not universally included in medical school curricula, we considered how graduates felt about the value of their mental health training to both their professional practice and personal life. To the best of our knowledge, our study is the first in Australia to consider the impact of this component on medical graduates.

## Method

### Participants

The interviews reported in this study were conducted as part of a mixed-methods research project. This paper focuses on the qualitative aspect of the project which aimed to ascertain how well the JMP prepared international medical graduates for practice in diverse health systems with reference to a range of clinical capabilities, including those related to patient-centered care. Ethics approval H-2018-0074 was granted by the University of Newcastle Human Research Ethics Committee (HREC).

A total of 120 international graduates of the JMP between 2010 and 2017 inclusive were contacted by the University of Newcastle (UoN) Alumni organization via email, and by social media messaging and posts, and invited to participate in the study. In the first part of the study, a 60-item survey^4^ asked questions about preparedness to practice relating to 9 domains and was administered to consenting participants using the cloud-based SurveyMonkey software, and participants were asked to indicate their willingness to further participate in a face-to-face interview. A response rate of 45% was received for the survey, of which 40.75% agreed to an interview. Selection criteria for the interview included all international graduates of the JMP program from 2010 to 2017 who were currently practicing within a healthcare system different from the one in which they received their training. The current article reports the results of the qualitative interviews.

Face-to-face interviewees indicated their willingness to participate by signing a consent form agreeing to an audio recording of their responses and to publishing of de-identified comments. Of the 40.75% (22) who agreed to interviews, 33.3% (18) of graduates were interviewed in the location of their employment within and outside Australia.

Interviews were structured using 18 standardized questions (Appendix 1) to elicit graduate perceptions of their preparedness to practice and reflections on their experience in the JMP program. Thirty minutes was allocated per interview with the capacity to extend if required. Interviews were conducted by 2 investigators, one who acted as facilitator, and one as note taker. Recorded interviews were transcribed, anonymized, and securely stored in accordance with UoN Research Data and Primary Materials Management Procedure.

### Analyze

An inductive thematic analysis^5,6^ method which consisted of a series of iterative steps was used. Initially, researchers transcribed audio recordings and familiarized themselves with the interviews, a process known as immersion in the raw data, until the content of each interview was well-known. The approach remained focused on the data, with each investigator independently coding a small number of interviews before meeting to reach a consensus on the use of codes. Regular meetings ensured that coding remained flexible and organic.

This was followed by identifying a thematic framework including key issues and concepts, which were then synthesized into common themes and organized as superordinate or subordinate. Finally, these themes were mapped and interpreted in relation to the original research question.

## Results

Eighteen graduates were interviewed; 11 were practicing in Malaysia, 5 in Singapore, and 2 in Australia. The majority (approximately 80%) of international students admitted to the JMP until 2017 were from Malaysia and Singapore. Results from the 18 interviews are presented in 2 ways: a summary in Table 1 and a comprehensive explanation of and evidence for superordinate and subordinate themes.

**Table 1.** A synthesis of the superordinate and subordinate themes that emerged from the 18 graduate participant interviews.

SUPERORDINATE THEME	SUBORDINATE THEME
Ahead of the curve	Holistic/patient-centered approach (communication, empathy, rapport) Mental health
Beyond books	Confidence and self-sufficiency Open-mindedness and adaptability Increased cultural sensitivity
Beyond borders	English language—hit and miss Bridging different health systems—boon or barrier? Returning local—culture shock Snakes and ladders Great expectations

Preamble: The interview will take approximately 45–60 min. In the interests of ensuring all interviewees are allocated equitable time, a response may be interrupted if interviewers feel time is running short.

All responses will be kept confidential. We ask that interviewees respond in a frank and an honest manner.

### Superordinate theme: ahead of the curve

Participants overwhelmingly reported that their undergraduate communication skills training and the patient-centered focus of the course had prepared them extremely well for future interaction with patients and colleagues, particularly in comparison to peers who had trained elsewhere. Participants who had been admitted to the JMP through selection interviews were supportive of the emphasis placed on communication skills even at that stage. Likewise, most participants regarded the undergraduate mental health training they received to be of significant value with respect to their personal health. These skills have proven beneficial in coping with stresses encountered in navigating their role as junior clinicians.

### Subordinate theme: holistic/patient-centered approach (communication, empathy, rapport)

When asked about the relevance of the JMP's patient-centered model, specifically the relevance to their practice as clinicians of training around the importance of empathy, rapport building, and interacting with patients, all graduates believed themselves well-prepared. Those practicing outside Australia believed themselves better trained in this domain than local graduates.

…we do this better than local grads. [Graduate (G) 7]

I really like how the JMP trained us… to treat the patients, to communicate with the patients, to understand the patients and I don’t see that's being practised here in Malaysia. (G6)

Fifteen participants noted that learning to establish rapport and respond empathically meant that, as PGY1’s, they felt comfortable and confident interacting with patients. The value of a holistic approach was also acknowledged:

…to manage the patient as a person not just their disease. (G14)

I think that in our career the empathy and understanding of the patient is very important because besides treating their diseases that's the most important aspect of being a doctor. (G3)

I think being empathetic to patients like we have in the training in Australia and the JMP gives me more advantages with the patients not just in my internship but in my other jobs as well. (G12)

The JMP does a good job in helping us to think from the patient's perspective to consider them as a person. Their mental health and spiritual health. (G7)

The JMP's interactional skills modules include considerable opportunity to engage with simulated and actual patients to discuss a range of physical and psychological health issues and a spectrum of sensitive topics, such as sexuality and suicidal thinking. Fifteen participants commented on the efficacy of these modules.

…the sessions we had …really apply to real life work. (G8)

…really useful – it's just natural interaction with the patients, you don’t have to think to do it, it's just there. (G17)

…comfortable breaking bad news, …comfortable in asking mental health questions and …also comfortable to ask questions about gender…. (G11)

All participants agreed the JMP interactional skills domains were advantageous regardless of the health system in which graduates practiced:

…the most important aspect of the JMP…learned. (G12)

The emphasis on aspects of communication and treatment such as shared decision-making was particularly noted as an asset by graduates who found themselves well-prepared for their return to countries and health environments in which considerable changes have taken place, such as increased patient literacy:

…nowadays most of the patients are very educated… they want to… be included in the decision…. (G15)

Moreover, the JMP patient-centered and communication focus was praised as instrumental in shaping graduates’ interaction with a growing role for the multidisciplinary team:

…multidisciplinary teams… so… very relevant to day-to-day practice. (G18)

### Subordinate theme: mental health

Our study considered how graduates felt about preparedness to implement their mental health training and the usefulness of this training to both their personal lives and professional practice. A theme was identified around how well the JMP mental health training equipped undergraduates with skills beyond professional implementation to focus on personal awareness, and utilization, of the various supports offered within the program and wider community.

Fifteen graduates identified that their training had been broadly valuable:

The JMP stresses that we open up everything that we need to…I’m quite comfortable expressing myself…to those I trust. (G7)

I am very comfortable talking about mental health issues. (G1)

It somehow shapes your mind to be positive… you have this kind of perspective I think it helps a lot. (G3)

The same 15 participants acknowledged comfort with discussing personal mental health issues in their home countries, albeit with caveats due to existing stigmas:

After studying the JMP I felt comfortable in discussing my personal issues with fellow students… Maybe not as much with the local grads, they’re not as open to discussing non-medical issues as they see them. (G13)

I’m actually very comfortable talking about it. I think I became quite open about it and a bit more sensitive towards it. Even among colleagues you can see some of them are stressed and we can easily talk to them where other people would just ignore them. (G15)

Newcastle prepared me to be more open about talking about mental health issues and not to be stigmatised by it. (G10)

However, graduates noted limited opportunity to implement their training in mental health in their clinical work, due to time pressures resulting from a high workload, and continuing stigma.

Compared to my other doctors who studied local it seems like they are not really into communication skills because in terms of Malaysia we have so many patients … so that's not the main concern … as long as you’re seeing patients and giving medication. But from where I’ve been teached it's important, it's part of the therapeutic relationship…. (G16)

There is a real attitude of ‘suck it up’ and ‘carry on’ in Singapore (G16)

The mental health side of things is still very taboo in a sense…. (G8)

Those graduates who had completed a psychiatry rotation as junior doctors claimed they were expected to concentrate on the “medical side of things” (G11).

Notwithstanding the lack of opportunity to apply their psychiatry training clinically, 6 graduates acknowledged a level of confidence in discussing mental health with patients when the possibility arose:

I’m comfortable in asking mental health questions to them and I’m also comfortable to ask questions about gender and stuff like that. (G11)

At the very least I can look at the patient, I can assess the patient and I can talk to the appropriate person that they need. (G2)

…it has helped me identify some patients that have a mental condition, and it makes me more aware…. (G4)

Graduates indicated that training in mental health had proven beneficial to eliciting important social history from patients. Moreover, this training helped prepare graduates to identify patients who had a mental health condition in association with chronic diseases.

…it has helped me identify some patients that have a mental condition, and it makes me more aware because some patients with chronic health diseases do have mental health problems. So, it makes you a bit more are that you would consider that when you are dealing with the patient's issues. (G4)

Importantly, despite barriers to implementing their psychiatry training clinically, most graduates acknowledged a level of confidence in discussing mental health with patients when the possibility arose. Overall, these responses indicate that despite graduates having limited clinical opportunity to apply their mental health training in their home countries, which were mainly Malaysia and Singapore, it was still considered of value as it had proven beneficial to maintaining personal mental health and to identifying important signs of mental distress in patients.

### Superordinate theme: beyond books

Most graduates highlighted particular skills and attitudes acquired beyond the set curriculum. Specifically, their learning experiences promoted professional and personal confidence. Furthermore, their training appeared to have facilitated greater open-mindedness, the ability to perceive things from different perspectives and increased cultural sensitivity, all of which they believed contributed to their preparedness for practice.

### Subordinate theme: confidence and self-sufficiency

Thirteen participants reported that the self-directed PBL model promoted confidence and independence as lifelong learners:

I think the JMP the first thing is they teach me to stand on my own, to find, research and everything rather than being spoon fed. (G15)

The freedom to let you follow and do what you think you need to do is strength. (G9)

Graduates attributed a broadening of their life skills to the learning environments and opportunities provided by the JMP.

I think the programme itself shapes you to be a better doctor… and everything so more like personal experience and how you handle your life. (G3)

The comparatively less hierarchical health system experienced by students in Australia was considered to give students the confidence to express opinions with both peers and more senior doctors.

Because I was exposed to Australia where the specialist and the other team members are quite close together and worked more as a team so the respect is more than fear… I brought that feeling and that understanding to Malaysia so I respect more than fear. (G5)

Sometimes I’m not wrong or right but I have the confidence to say it out loud. Because in the JMP we actually appreciate all the opinions of others. (G12)

### Subordinate theme: open-mindedness and adaptability

Fifteen graduates described greater open-mindedness, the ability to perceive things from different perspectives and recognize the need to see things from a broader view. They reported greater adaptability. Graduates attributed developing these qualities to the learning environments and opportunities provided by the JMP.

We become more open minded because we’ve been exposed to another system. (G11)

I think I’ve become more open minded… when you’re exposed to another system you become more flexible. (G5)

…the JMP makes you more confident and…the JMP helps you be flexible to adapt to any placement…. (G12)

We are more adaptable to things, if we know that something can be done better another way, we are more efficient at doing that. We are probably better at accepting changes. (G12)

### Subordinate theme: increased cultural sensitivity

Ten participants reported their training prepared them to be more aware of, and sensitive towards, the cultural differences which exist in their diverse communities. They noted that exposure to First Nations peoples and their health challenges in Australia had supported this learning.

… we do have a variety of races. And a variety of cultures. So, in that sense the relevant thing is to respect each culture individually so that was something that was taught well in the JMP where we had to learn to respect the Indigenous population and to understand that their culture is a bit different. (G7)

…helped because rural and Indigenous helped me appreciate how different cultures and how health is impacted by all these other factors. (G6)

…the local Malay are more disadvantaged in certain aspects of their health because of their culture, what they eat, how they approach their general physical and mental health, so it gives you some help in understanding from what we learned about Indigenous culture in Australia. (G9)

### Superordinate theme: beyond borders

Participants highlighted the advantages and disadvantages of being immersed in a different cultural and learning environment. Recurrent themes were identified around English being the dominant language of professional communication, the impact of training and working in a different health system, and the effect of these on transitioning to local issues and expectations.

### Subordinate theme: English language—hit and miss

Nine participants believed that becoming proficient in English during their stay in Australia had been a distinct advantage in effectively communicating professionally:

We are expected to read and convey all the particular things about the patient in English. (G3)

…in terms of discussion between the doctors and doing rounds and presenting cases to the bosses because all of those happen in English. (G5)

I found that people who trained in Indonesia, especially, because they use Indonesian terms, struggle a lot with it. (G10)

Studying in English with the medical terms {made it} really easy to adapt to our system during internship. (G10)

This was especially so given that “reports and referrals are all done in English” (G11).

Six participants noted that while fluency in English was advantageous in professional communication, language barriers remained for some patient interactions, particularly with the older generations and in more remote regions. Training in an English-speaking country might have come at the cost of opportunities to become fluent in local dialects.

…patients here speaking different dialects – there's Malay patients, there's Indian patients’ so in terms of communicating with them there's still that barrier…. (G4)

In the big hospitals they speak English but here in a regional hospital they don’t. And…in local community clinic they don’t use English at all. (G15)

It's just for the older generation that we have to change our language to dialects which I’m not very fluent at yet. (G7)

### Subordinate theme: bridging different health systems—boon or barrier?

Sixteen participants agreed that having trained in Australia's health system had been advantageous. They believed this experience had broadened their perspective, and their confident knowledge of this health system, with its strengths and weaknesses, motivated them to seek opportunities for improvements to their own country's health system.

We can take the good sides of the Australian system and we can insert it into our Malaysian system so it's good. We can compare it and we can suggest how to improve it. (G13)

… based on different systems we can learn what are the pros and cons of each system, so to me if you can bring the good of each system and bring them together. (G14)

I think you learn quite a bit and what you learn you can bring back from that other country. (G4)

When asked about any disadvantages of studying in a different healthcare system to that of their home country, 12 of 16 respondents mentioned that navigating the unfamiliar administrative requirements posed challenges. It was also commonly reported that supervisor expectations were perceived as more demanding than in Australia, and they felt relatively underprepared for this more hierarchical style of interaction. Another drawback of not training in their own country is related to differences in the epidemiology of diseases between Australia and their country.

It took a good 50% of my internship period to get used to the system. For example, learning what services are available for patients. How to go about ensuring that a patient can afford a particular health service. (G15)

And also, the expectation of people is different as well. It's different in so many things in terms of patients, in terms of your superior, in terms of your colleagues…. (G3)

And the spectrum of diseases you see, that was a big challenge. (G12)

### Subordinate theme: returning local—culture shock

For some graduates, returning to their home countries and adapting to the work culture of their local system proved challenging. They found themselves having to realign their approach to patient interaction as well as reassess how they might meet local system expectations. Nine participants highlighted their difficulty adjusting to the business models of their health system and to the limited opportunities for patient interaction and rapport building.

Our superiors don’t really appreciate if you talk too much or spend too much time with the patient. They like to see you work. (G13)

…in this Asian context. It's very much a business model view, so the more patients you clear and discharge, the more you earn and the better for the economy and the hospital. So, it's really very different here. (G4)

…there's one limitation – time constraint. I can’t spend more time to explain about his/her disease in the time so it's a bit challenging. So in that time, when there is time constraint I can’t build a rapport.(G15)

### Subordinate theme: snakes and ladders

Many participants believed that the collaborative clinical training model they experienced equipped them with the skills to engage confidently with both peers and superiors. However, this was undermined for some, with 8 participants commenting on the existence of a distinct hierarchy in the local health system and its impact on their experience, a contrast to the more informal system they had encountered during their training in Australia.

…how you address your superiors is different. Australia is quite laidback whereas in Malaysia the hierarchy is important. (G12)

I think if I’d only been in the Malaysian system the relationship between me and consultants would be more of fear rather than respect. Because I was exposed to Australia where the specialist and other team members … worked more as a team … the respect is more than fear. (G5)

Back in Australia you can express your opinion … But here it's different – it's all about the hierarchy, so you have to be really careful about what you discuss with them. (G11)

… if you tried to voice up that would be a disadvantage in Malaysia. (G14)

### Subordinate theme: great expectations

The disparity of expectations between seniors and consultants locally and in Australia was of significant concern to 13 graduates. This concern focused on the perceived expectation that interns be capable of working autonomously and with a level of expertise for which they felt themselves unprepared.

…when you are in Malaysia you already have to know what to do on your own. Even though your bosses will give you plans … you have to think on your own and sometimes you have to execute it first before informing your doctor (senior)… So we have to act first. So, expectations are different. (G5)

…so we’re managing the patients ourselves rather than with the help of the registrars. And that's for overnight admissions and so there's a lot of like responsibility. So, I think in terms of knowledge there's a lot more expected of us in terms of how to manage patients. (G10)

… in terms of more practical like taking bloods, IV lines … in Malaysia that's what we are expected to do in our housemanship. On our first day of working they are expecting us to be expert already. (G17)

While all participants acknowledged the many benefits of their Australian training in preparing them for practice, they were also forthright in raising the several challenges they faced in adapting to markedly different health systems.

## Discussion

Overall, the results of this study indicate that International JMP BMed graduates perceived themselves to be adequately prepared for work as first-year medical graduates in their place of internship. Participants uniformly acknowledged the advantages of the JMP's strong emphasis in the domains of communication training and skills, self-directed learning, and mental health. However, they identified a relative lack of work-ready clinical skills to be a primary shortcoming of their training.

Contrary to concerns raised by Adelaide graduates to Anna Chur-Hansen in 2004^3^ that Australia's “patient-centered, egalitarian medical communication skills…may not be appropriate in Malaysia,” international JMP graduates, a decade later, believed the importance afforded communication skills in both the JMP's student selection process and curricula as justified and relevant. Similar to previous findings graduates cited interactional skills, specifically development of rapport and ability to convey empathy, to be not only pertinent to their interaction with patients, but core to providing holistic treatment.^7^ Moreover, they evaluated the JMP as having provided interactional skills superior to those of their counterparts who trained elsewhere.^8^ These skills contributed to greater personal and professional confidence. Some decades ago research identified Newcastle graduates as rating better in the areas of interactional skills and self-directed learning than their counterparts who trained in more “traditional” medical schools in Australia.^9,10^ Interactional skills training and problem-based learning are now included in every Australian medical curriculum, but this may not be the case for medical schools in Malaysia and^8^ Singapore which may continue to offer a more traditional approach. In this respect, this previous research may be relevant to the findings we report.

The variety of practice cases in communications skills teaching ensured students engaged with a spectrum of general and sensitive presentations, and the results of this study suggest that students did indeed develop confidence in communicating with patients on sensitive topics. The international graduates’ responses indicate their communication skills training equipped them to be “work-ready plus”^8^ having fostered the emergence of a patient-centered professional identity. Respondents noted a move in their own health systems towards more patient-centered care. This shift is supported by a Southeast Asian study in which participants expressed general preference for a partnership style of doctor–patient communication, where trust, equality, and reciprocal exchange of information and participation featured prominently.^11^ Graduates recognized this aspect of training as integral to preparing them well for returning home to hospital systems transitioning to “practice in partnership.”^12^ This partnership extends beyond patients to communicating confidently with senior clinicians and multidisciplinary teams.

Given that higher education leaders seek to produce graduates who possess capabilities that are relevant for future workplace requirements, not just current needs,^8^ the JMP interactional skills domains were perceived as advantageous regardless of the health system in which graduates practiced.

Given the evidence of high levels of psychological distress in junior doctors,^13,14^ the findings in this study, that graduates found training in mental health to be beneficial not only in treating patients but for maintaining personal wellbeing, underscore the importance of mental health curricula. The JMP mental health training aims to optimize medical student wellbeing^15^ by equipping undergraduates with skills beyond professional implementation to focus on personal awareness and utilization of appropriate supports. Previous research has suggested that medical students and doctors are reluctant to seek help^16,17^ so the increased willingness of our graduates to discuss psychological issues with trusted others is encouraging.

Recognizing the broader Australian approach to mental health issues has been quite different from that of countries from which many international students originate, of interest was whether their JMP training had been of value in diverse contexts and cultures. Stigma may be more acute in some cultural groups and communities.^15^ Studies conducted in Singapore have reported that the stigma towards mental illness is harsher when compared to the Western world, attributing this to various reasons including greater conservativism of Asian cultures,^18^ associating mental illness with stigma, shame and loss of “face,”^19^ and mental weakness.^20^ Participants in our study agreed that there was stigma and suggested similar explanations. A Malaysian study suggested that stigma can be amplified by religious beliefs and expectations noting “followers of Islam are susceptible to the misperceptions that mental illness arises from ‘supernatural activities’”^21^: a religious element was not suggested by our study participants.

Apparent additional benefits of the JMP communication and patient-centered curriculum included greater open-mindedness, the ability to perceive things from different perspectives and recognize the importance of taking a broader view.

Participants noted their preparedness to assert confidence in their abilities. This confidence was attributed to the PBL environment which encourages self-directed and collaborative learning as opposed to the didactic approach participants had experienced in more traditional educational settings and as pre-tertiary students. Unlike students in their home countries who are very deferential towards tutors as authority figures, fear confrontations with these authority figures, and tend to be more dependent learners,^22^ the comparatively less hierarchical health system experienced by students in Australia was noted as a strength. This enabled graduates to engage more fully in their learning and offered them a voice with both peers and seniors as they progressed and in their eventual work environment. This represents a clear shift in attitude and confidence from returning Malaysian graduates in 2004 who found the hierarchical system challenging and unfriendly and were also concerned about seniors and the way (they) might be treated by them.^3^ However, even in our study, it was common for more senior or supervising doctors to be referred to as “superiors.”

Whereas Malaysian graduates from Adelaide returning to their home country in 2004 “expressed concern that having learnt medicine in English, they may have communication difficulties when trying to speak to their Malaysian patients,”^3^ recent JMP graduates who returned to Malaysia and Singapore believed studying in English to be, overall, advantageous. While translating medical terminology into local dialects remains a challenge for graduates practicing in more regional or rural areas, English is widely spoken in international communities and a main language of communication professionally.

Graduates perceived additional challenges in needing to adapt to the local health system, master language dialects, cope with greater clinical responsibility, a more hierarchical structure, and longer hours.

Significantly, graduates felt that their exposure to the Australian healthcare system and approaches to medical education had given them a valuable perspective that they could share. Coupled with their greater confidence in communicating their own views and opinions, they expressed optimism about the potential for these shared insights to contribute to improvements in the health systems in the countries in which they were now practicing.

Graduates also identified some apparent weaknesses in their Australian medical undergraduate course. They noted that the self-directed focus of their training, at times, lacked structure. It seemed to them that they had had less clinical exposure relative to local graduates. They particularly felt less practiced at common bedside clinical procedures, administrative requirements, and clinical governance.^23^ The graduates perceived their local counterparts to be more practiced and proficient as a result of more patient interactions and autonomy to participate in hands-on clinical procedures. Apart from weighting in the curriculum, broader factors may limit opportunities for medical students in Australia to practice procedural skills with real patients. There is often a higher ratio of students per patient and protocols around the scope of practice tend to pose significant limits on procedures medical students may undertake, even under supervision. Participants also felt that they had achieved a less proficient level of knowledge around pharmacotherapy, which may reflect particularities of the Newcastle program, rather than being generalizable to Australian medical schools.

Our findings have both local and more general implications. The JMP program may benefit from a greater emphasis on opportunities for “hands-on” clinical experience, particularly the everyday minor procedural skills required of interns. It may also be advantageous for medical schools that enrol international students to create opportunities for those students to gain experience working in the health systems of their home countries before returning to work there as a junior doctor. This could be achieved through elective opportunities.

This study offers several strengths that enrich its contribution to existing literature. Primarily, it provided a platform for international medical graduates to critique the JMP from their unique perspective. Secondly, it addresses some of the challenges students face in applying their mental health knowledge and skills in diverse cultures with distinct obstacles. Furthermore, the study sheds light on how their understanding of mental health influences their self-care practices, an aspect not previously explored in depth.

However, there are notable limitations to consider. The response rate of 33.3% was lower than anticipated. This was influenced by the fact that many graduates, who had traditionally returned to their home countries post-graduation, were interning in New South Wales (NSW) and the study aimed to assess preparedness in a different healthcare system. The transferability of the findings may also be limited by the uneven representation of students from different countries, with a disproportionately high number of Malaysian and Singaporean graduates potentially skewing the results. Additionally, the study's reliance on graduates from a single university may raise questions about the generalizability of the findings.

## Conclusion

Our study identified key strengths of the JMP, primarily patient-centered care, self-directed and team-based learning, and communication skills, as contributing to confidence, open-mindedness, and self-sufficiency in professional and personal domains. The program's emphasis on mental health was seen as beneficial to both personal wellbeing and patient care.

Despite acknowledged strengths, there were areas in which the program was less successful in preparing graduates. There is a need for opportunities to master common procedures and a greater focus on assessment, treatment planning, and clinical handover. Additionally, challenges related to the transnational location of the program pose specific difficulties.

A notable aspect is the commitment of graduates to implement what they considered the best elements of the Australian health system in their local contexts, showcasing a positive exchange of knowledge and practices.

Ultimately, promoting cultural sensitivity and awareness of different health systems better prepares international graduates’ entry to a diversity of workforces.

## Supplemental Material

sj-docx-1-mde-10.1177_23821205241272360 - Supplemental material for Bridge to Practice: A Qualitative Evaluation of Joint Medical Program (JMP) International Medical Graduates Perceived Preparedness for Professional Practice Supplemental material, sj-docx-1-mde-10.1177_23821205241272360 for Bridge to Practice: A Qualitative Evaluation of Joint Medical Program (JMP) International Medical Graduates Perceived Preparedness for Professional Practice by Michelle Foot, Khanrin Vashum, Pavana Ballal and Lisa Lampe in Journal of Medical Education and Curricular Development

sj-docx-2-mde-10.1177_23821205241272360 - Supplemental material for Bridge to Practice: A Qualitative Evaluation of Joint Medical Program (JMP) International Medical Graduates Perceived Preparedness for Professional Practice Supplemental material, sj-docx-2-mde-10.1177_23821205241272360 for Bridge to Practice: A Qualitative Evaluation of Joint Medical Program (JMP) International Medical Graduates Perceived Preparedness for Professional Practice by Michelle Foot, Khanrin Vashum, Pavana Ballal and Lisa Lampe in Journal of Medical Education and Curricular Development
