# Supplementary material for: Bridge to Practice: A Qualitative Evaluation of Joint Medical Program (JMP) International Medical Graduates Perceived Preparedness for Professional Practice
Source: J Med Educ Curric Dev. 2024 Aug 16;11:23821205241272360. doi: 10.1177/23821205241272360 (PMC11329892; doi:10.1177/23821205241272360)
Supplement: sj-docx-1-mde-10.1177_23821205241272360 - Supplemental material for Bridge to Practice: A Qualitative Evaluation of Joint Medical Program (JMP) International Medical Graduates Perceived Preparedness for Professional Practice [file sj-docx-1-mde-10.1177_23821205241272360.docx]

*Preamble: The interview will take approximately 45 – 60 minutes. In the interests of ensuring all interviewees are allocated equitable time, a response may be interrupted if interviewers feel time is running short.*

*All responses will be kept confidential. We ask that interviewees respond in a frank and honest manner.*

**INTERVIEW QUESTIONS**:

1. Do you think you were better than or as prepared as peers who trained elsewhere? If yes, what advantage do you think the JMP provided? If no, why not?

2. What differences did you experience in the level of support provided by your supervisors and senior clinicians during your JMP training and the year you commenced work?

3. Can you provide examples of feedback you were given about meeting or not meeting the expectations of the senior clinicians during the year you commenced work?

4. How relevant is the JMP patient-centred model to your current practice and experience?

5. Has the JMP emphasis on the importance of empathy/rapport building/communication been relevant to your practice?

6. Are these areas prioritised in your place of commencing work? Why/why not?

7. How has the Mental Health component in the JMP been of use in your practice location? For example, is this a feature of history taking where you practise?

E.g. IMU students have no introduction to mental health prior to arrival as per JMP Year 2

8. In relation to the areas of Rural and Indigenous health – how relevant or useful has this been to your practice?

9. What suggestions can you make to better prepare international students for transnational employment?

10. Did the global health component of the JMP course adequately prepare you for the medical presentations you encountered during the year you commenced work?

11. Are there any ethnicity and/or gender biases in your country of practice? Please elaborate on the impact of these biases to your practice.

12. Were/Are there any rotations for which you felt more prepared than others? What factors influenced this?

13. On completion of the JMP how comfortable did you feel about discussing personal stresses/mental health issues?

14. What do you perceive as the shortcomings or deficiencies of the JMP in preparing students for their first year of work?

15. What do you perceive as the strengths of the JMP in preparing students for their first year of work?

16. In what ways has studying in English impacted your ability to effectively understand and communicate medical terminology in your country of origin?

17. What do believe are the disadvantages to studying in a different healthcare system to that of your own?

18. What do believe are the advantages to studying in a different healthcare system to that of your own?
